# Determination of the *In Vitro* and *In Vivo* Antimicrobial Activity on Salivary Streptococci and Lactobacilli and Chemical Characterisation of the Phenolic Content of a *Plantago lanceolata* Infusion

**DOI:** 10.1155/2015/286817

**Published:** 2015-02-15

**Authors:** Gianmaria Fabrizio Ferrazzano, Tiziana Cantile, Lia Roberto, Aniello Ingenito, Maria Rosaria Catania, Emanuela Roscetto, Giuseppe Palumbo, Armando Zarrelli, Antonino Pollio

**Affiliations:** ^1^Department of Neurosciences, Reproduction and Odontostomatologic Sciences, University of Naples “Federico II”, Via Pansini 5, 80100 Naples, Italy; ^2^Department of Molecular Medicine and Medical Biotechnology, University of Naples “Federico II”, Via Pansini 5, 80100 Naples, Italy; ^3^Department of Chemical Sciences, University of Naples “Federico II”, Via Pansini 5, 80100 Naples, Italy; ^4^Department of Biology, University of Naples “Federico II”, Via Pansini 5, 80100 Naples, Italy

## Abstract

*Introduction.* Plant extracts may be suitable alternative treatments for caries. *Aims*. To investigate the *in vitro* and *in vivo* antimicrobial effects of *Plantago lanceolata* herbal tea (from flowers and leaves) on cariogenic bacteria and to identify the major constituents of *P. lanceolata* plant. *Materials and Methods.* The MIC and MBC against cariogenic bacteria were determined for *P. lanceolata* tea. Subsequently, a controlled random clinical study was conducted. Group A was instructed to rinse with a *P. lanceolata* mouth rinse, and Group B received a placebo mouth rinse for seven days. The salivary colonisation by streptococci and lactobacilli was investigated prior to treatment and on the fourth and seventh days. Finally, the *P. lanceolata* tea was analysed for its polyphenolic content, and major phenolics were identified. *Results and Discussion. P. lanceolata* teas demonstrate good *in vitro* antimicrobial activity. The *in vivo* test showed that Group A subjects presented a significant decrease in streptococci compared to Group B. The phytochemical analysis revealed that flavonoids, coumarins, lipids, cinnamic acids, lignans, and phenolic compounds are present in *P. lanceolata* infusions. *Conclusions. P. lanceolata* extract could represent a natural anticariogenic agent *via* an antimicrobial effect and might be useful as an ancillary measure to control the proliferation of cariogenic flora.

## 1. Introduction

Although the prevalence of dental caries has been slowly declining in many developed countries, this disease continues to be a major public health problem in industrialised countries and has become an emerging public health problem in developing countries [1].

Various preventive measures have been attempted to control the insurgence of dental caries, such as topical or systemic fluorides, fissure sealants, and dietary approaches, but they are not sufficient to control the disease, which has a multifactorial aetiology [2].

Bacteria belonging to different species of* Streptococcus* and* Lactobacillus* play a lead role in the development and progression of caries [3]. Because these microorganisms are the most prominent factors in dental caries process, reducing their level in the oral cavity will provide an additional rationale for the prevention of dental caries [4].

Chlorhexidine is an antimicrobial agent clinically efficient against a wide range of cariogenic microorganisms [5]. However, the use of chlorhexidine for caries prevention has been a controversial topic among dental educators and clinicians, due to several undesirable side effects, including the formation of extrinsic stain on the tooth and tongue and emergence of bacterial resistance [6]. Within this context, alternative antimicrobial treatments, such as antibacterial compounds extracts from plants, reducing cariogenic microflora salivary counts, can be proposed to control this disease [7–10].

The genus* Plantago* (Plantaginaceae) encompasses approximately 275 species with a cosmopolitan distribution. Recent studies have confirmed that some* Plantago* species have considerable antiviral, anti-inflammatory, and antioxidant activities [11–13]. Phytochemical studies have also shown that the genus* Plantago* contains a great amount of phenolic compounds (flavonoids and tannins). In particular, phenolic compounds seem to play a potential role in the control of bacterial growth and could prevent tooth decay, decreasing growth, and virulence of pathogenic oral flora [14]. A recent study has reported that* P. lanceolata* methanol/water extracts are rich in phenolic acids, with the benzoic acid derivatives hydroxybenzoate and 3,4,5-trihydroxybenzoate (gallic acid) being the most highly represented [11].

Hot infusions (herbal teas) represent by far the most popular type of consumption of herb to which medicinal properties have been acknowledged in traditional medicine [15]. Tsai et al. (2008) have demonstrated that several herbs commonly used as tea in Taiwan exhibited anticariogenic and anti-inflammatory properties [16]. According to European Medicines Agency (EMA) [17], herbal tea made from* P. lanceolata* leaves is used in European countries as a demulcent for the local treatment of oral or pharyngeal irritations and is considered a safe preparation with no known contraindications. In this study, an* in vitro* assessment of the antimicrobial activity of* P. lanceolata* tea against cariogenic bacterial strains of the species* Streptococcus* and* Lactobacillus* isolated from clinical samples was performed; subsequently, a mouth rinse made with an infusion of dried* P. lanceolata* leaves and flowers was tested* in vivo* for its effectiveness in reducing cariogenic microflora salivary counts. Finally, the* P. lanceolata* herbal tea was analysed for its polyphenolic content, and major phenolics were identified.

## 2. Materials and Methods

### 2.1. Chemicals and Reagents

Phenolic compound standards were purchased from Sigma Aldrich (Milan, Italy). All the other chemicals and solvents were purchased from Fluka (Saint-Quentin Fallavier, France) at analytical or HPLC grade and were used as received.

### 2.2. Plant Material and Extract Preparation

*P. lanceolata* was collected in June 2012 in the Royal Park of Capodimonte, Naples, Italy. The voucher specimen (no. 0134) was prepared and identified in our lab and deposited at the Herbarium of the Botanical Garden of the “Federico II” University in Naples, Italy.

Flowers, leaves, and roots of* P*.* lanceolata* were dried separately at 55°C for 48 h in a Carlo ErbaE.28 CL.1 Oven and then ground to a coarse powder. A total of 2 g of powder of each part of the plant was suspended in 20 mL of* Amorosa* water (still mineral water, low in sodium, and ideal for the preparation of baby and toddler food) at 100°C for 3 min. The preparation was infused for 8 min and then was cooled to room temperature, filtered, and evaporated to complete dryness.

To prepare the mouth rinse, 200 g of pulverised material (flowers and leaves) was suspended in 2.0 L of* Amorosa* water following the same procedure previously described. The preparation was filtered, placed in a sterilised 3.0 L glass container, and stored at 4°C before use.

### 2.3. *In Vitro* Antimicrobial Tests

The bacterial strains used for testing were* L. casei, S. bovis, S. mutans, S. mitis, S. parasanguinis, S. viridans*, and* S. sobrinus*. The strains were from clinical specimens obtained at the Diagnostic Unit of Bacteriology and Micology of the University of Naples “Federico II.” Bacteria were grown on Trypticase Soy Agar with 5% Sheep Blood (TSS; Becton Dickinson, USA) plates at 37°C in 5% CO_2_ for 48 h.

The minimum inhibitory concentration (MIC) was measured using the standard microdilution method in 96-well polystyrene plates using brain-heart infusion (BHI) medium. The starting inoculum was 5 × 10^5^ CFU mL^−1^, and the final concentrations of the* P. lanceolata* infusion ranged from 4 to 0.025 mg mL^−1^. To determine the minimal bactericidal concentration (MBC), 50 *μ*L of bacterial suspension from the wells containing extract concentrations equal to or higher than the MIC were inoculated in 5 mL of sterile BHI medium and incubated for 24 h at 37°C under a 5% CO_2_ atmosphere.

### 2.4. Biologic Assays on Human-Lymphocytic Cells

We have chosen two human cell lines one p53 negative and one p53 positive.

The p53-null U937 human leukemic monocyte lymphoma and the p53-positive isogenic human lymphoblast TK6 cell lines were obtained from American Type Culture Collection (Rockville, MD). Both cells lines grew in Dulbecco's Modified Eagle Medium, 2 mM l-glutamine, 100 *μ*g/mL streptomycin, 100 units/mL penicillin, and 10% foetal calf serum (FCS). All media and cell culture reagents were purchased from Life Technologies (San Giuliano Milanese, Italy).

Cell viability was assayed by using the Cell Proliferation Kit II (XTT, Roche, Milan, Italy). This assay is based on the cleavage of the yellow tetrazolium salt XTT to form an orange formazan dye by metabolic active cells; therefore, this conversion only occurs in viable cells.

The plant extracts were provided to our laboratory in the relative extraction solvents having all a nominal concentration of 50 mg/mL. The extracts were dried under vacuum and redissolved in DMSO 10% in water. Samples were stored at −30°C until use. Before measurements, samples were brought to room temperature under agitation and added to culture media in a ratio 1 to 10. In detail, 90 *μ*L of suspensions of U937 or TK6 cells (containing ~1 × 10^4^ cells in complete medium) were seeded into 96-well plates. Then, 10 *μ*L of each extract (50 mg/mL in 10% DMSO) was added to each well so that the final extract concentration was 5 mg/mL, while the DMSO content was reduced to 1%. Cells were incubated in these conditions at 37°C for 24 h in 5% CO_2_ atmosphere. Triplicate samples were prepared for any individual condition. As a positive control for cytotoxicity we used Triclosan at low concentration (0.03% as compared with 3% used in toothpastes). This synthetic is a polychloro-phenoxy phenol endowed with antibacterial and antifungal properties. For these reasons it is currently largely used in oral hygiene as additive of toothpastes to prevent gingivitis.

### 2.5. *In Vivo* (Clinical) Antimicrobial Tests

The clinical studywas conducted on a sample consisting of forty-four adolescents (24 males and 20 females) enrolled in the Department of Paediatric Dentistry at the University of Naples “Federico II”, Italy, ranging from 12 to 18 years old.

The inclusion criteria were good general health (ASA I: Healthy person; ASA II: Mild systemic disease) and an agreement to comply with study procedures.

Subjects who had used antibiotics or mouth rinses during the 14 days prior to the beginning of the study were excluded.

Only 2 subjects of the 44 were excluded for this reason. However, during the research period, 2 subjects chose to end their participation; thus, 40 subjects completed the entire protocol. Participation in the study was voluntary. All of the parents gave written informed consent after receiving verbal and written explanations of the experimental protocol and study aims. The protocol was approved by the Ethical Committee of the School of Dentistry, University of Naples “Federico II”, Italy.

A controlled random clinical study was conducted. The subjects were divided into 2 groups of 20 subjects, respectively (Group A and Group B). Patients were randomly assigned to test and control groups using blocked randomisation from a computer-generated list.

Salivary counts of* S. mutans* and* L. casei* were estimated using a chair-side test that contained 2 agar surfaces. The blue mitis-salivarius-agar with bacitracin was used to detect the presence of streptococci, whereas the light culture medium, Rogosa agar, was used to evaluate that of lactobacilli.

No special dietary restrictions were imposed on the subjects, and no tooth brushing was allowed for at least 1 h after consuming lunch and dinner. All of the subjects were encouraged to maintain their normal oral hygiene habits. All subjects were given the same brush and tooth-paste for daily oral hygiene.

Saliva was inoculated on a dip slide with selective media for streptococci and lactobacilli. After adding a NaHCO_3_ tablet to the tube, the dip slides were immediately cultivated at 37°C for 48 h. The tablet releases CO_2_ on contact with moisture, creating favourable conditions for bacterial growth.

The colonies were identified using a stereomicroscope with ×10 magnification, and the culture density (CFU/mL) was visually compared with the aid of a chart provided by the manufacturer. Bacterial colonies were categorised as low (<10^5^ CFU/mL of saliva) or high (> or = 10^5^ CFU/mL).

Two different mouth rinse formulations were prepared.

(1) Experimental mouth rinse (Group A): an experimental mouth rinse was prepared with an infusion of* P. lanceolata* leaves and flowers, as previously described.

(2) Placebo mouth rinse (Group B): the placebo mouth rinse was prepared with* Amorosa* water, coloured with food dye.

*Experimental Design*. During the experimental period, after collecting the first saliva sample (T0), all of the Group A participants were asked to rinse with 10 mL of the experimental mouth rinse; instead, the participants of Group B were instructed to rinse with 10 mL of a placebo mouth rinse that did not contain phenolic substances, for 60 seconds after performing oral hygiene, 3 times a day (after breakfast, after lunch, and before sleeping) for 7 days.

On the 4th (T1) and 7th (T2) days of treatment, additional salivary samples were collected and immediately incubated to calculate the density (CFU/mL) of* S*.* mutans* and* L*.* casei* for each subject. 

*Statistical Analysis*. At the end of the treatments, the data were processed using the Statistical Package for Social Sciences (version 10.0, SPSS Inc., Chicago, Illinois, USA). A regression binary logistic analysis was performed. The statistical significance level was established at *P* < 0.05.

### 2.6. Analysis of Phenolics

Infusions of* P*.* lanceolata* whole plants and separated parts (flowers, leaves, and roots) were subjected to different chemical tests to detect the presence of flavonoids [18], coumarins [19], lipids and cinnamic acids [20], and tannins [21].

Column chromatography (CC) was performed on a Merck Kieselgel 60 (230–400 mesh). HPLC was performed on a Shimadzu LC-10AD using a Shimadzu RID-10A UV-VIS detector. A semipreparative HPLC was performed using an RP18 (LiChrospher 10 *μ*m, 250 × 10 mm i.d., Merck) column with a flow rate of 1.2 mL min^−1^, using an injection volume of 200 *μ*L and a binary mixture of water/formic acid 0.1% (99 : 1) and methanol (solvents A and B, resp.). The gradient was as follows: 0 min, 70% A/30% B, reaching 35% A/65% B after 12 min, then reaching 100% B after 25 min, which was maintained for 12 min. A 10 min equilibration time was used between analyses.

^1^H- and ^13^C-NMR spectra were recorded on a Varian INOVA-500 NMR instrument (^1^H at 499.6 MHz and ^13^C at 125.62 MHz), referenced with deuterated solvents (CDCl_3_ or CD_3_OD) at 25°C. Proton-detected heteronuclear correlations were measured using a gradient heteronuclear single-quantum coherence (HSQC), optimised for ^1^ *J*_HC_ = 155 Hz, and a gradient heteronuclear multiple bond coherence (HMBC), optimised for ^*n*^ *J*_HC_ = 8 Hz.

MS spectra were obtained using a HP 6890 spectrometer equipped with an MS 5973 N detector. HR-ESI-MS/MS was performed using a Q-TRAP model API-2000 LC-MS/MS system equipped with a heated nebuliser source and using the Analyst software (Applied Biosystems).

### 2.7. Determination of Total Phenolic Content

Total phenolic content was determined spectrophotometrically according to the Folin-Ciocalteu's method with slight modifications [22]. Briefly, 1.0 mL of properly diluted samples, calibration solutions, or blank solution were pipetted into separate test tubes (20 mL) and 1.0 mL of Folin-Ciocalteu's reagent was added to each. The mixture was mixed well and allowed to equilibrate. After exactly 10 min, 10 mL of milliQ water and 3.0 mL of Na_2_CO_3_ solution (*w* = 5%) were added and the volume was made up with milliQ water. The mixture was swirled and put in a water bath at 40°C for 20 min. Then, the tubes were rapidly cooled on ice, and the colour generated was read at its maximum absorption. The measurements were compared to a standard curve of prepared gallic acid solutions (50, 100, 150, 250, and 500 mg L^−1^) and expressed as milligrams of gallic acid equivalents. All measurements were performed in triplicate, the absorbance was measured in 1 cm cuvettes, and for calibration solutions and blank preparation, a methanolic solution at the same concentration as the samples was used.

## 3. Results

### 3.1. Determination of* P. lanceolata* Infusion Phenolic Content and Identification of Major Constituents

A preliminary analysis was performed to identify the major constituents of the whole* P. lanceolata* plant. Flavonoids, coumarins, lipids, cinnamic acids, lignans, and phenolic compounds are present in infusions of* P. lanceolata *(Table 1).

**Figure 1:**
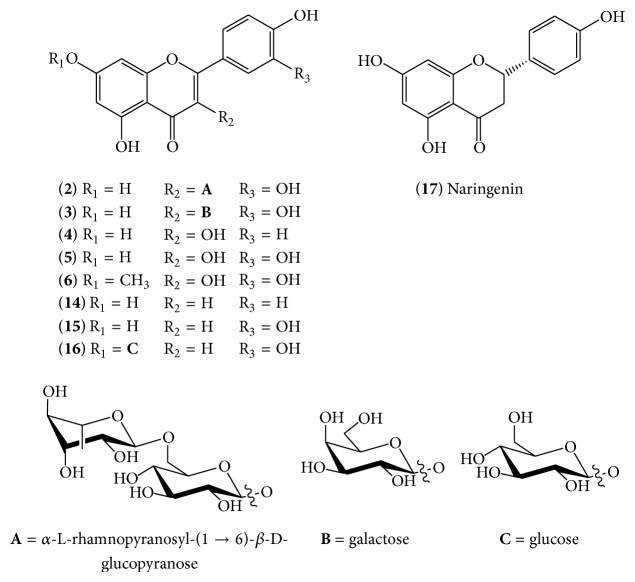
General structures of flavonoids isolated from* P*.* lanceolata*.

**Figure 2:**
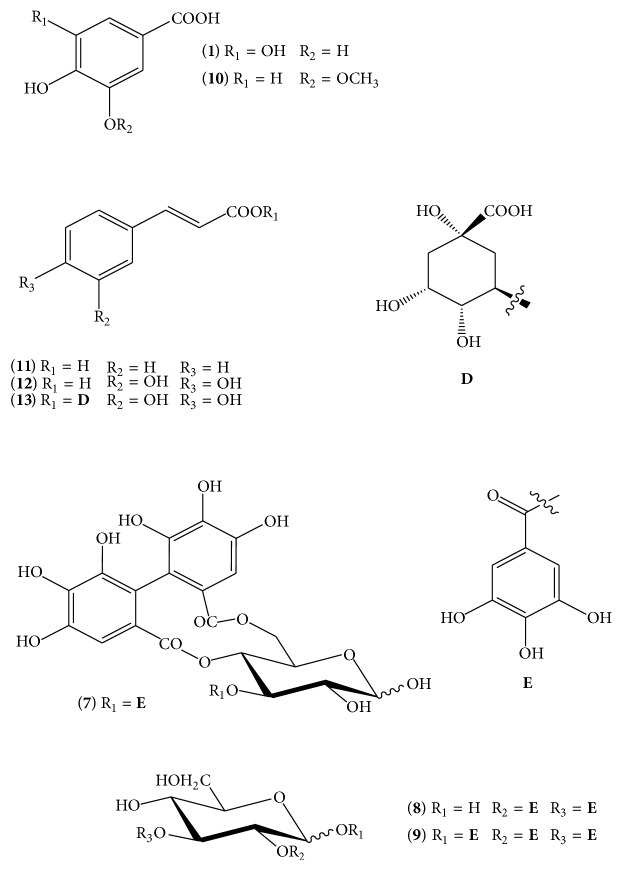
Cinnamic derivatives of* P*.* lanceolata*.

The hot water infusion of the entire* P*.* lanceolata* (flowers, leaves, and roots) contains different classes of phenolics, including flavonoids, coumarins, lipids, and cinnamic acids at a range of concentrations (Table 2). In the lyophilised infusion, the level of phenolic compounds was 40.5 ± 1.3 mg GA equiv/g DW, as revealed by HPLC analysis. The level of total phenolics, as detected by Folin-Ciocalteu's method, was higher (89.0 ± 2.5 mg GA equiv/g DW); the difference can be explained by the presence of protein, ascorbic acid, or reducing sugars in* P*.* lanceolata*, which contribute to higher Folin-Ciocalteu values. An LC/MS/MS technique was applied to the hot infusion prepared from* P. lanceolata* leaves (yield of 5.4% of the air-dried raw material mass), leading to the isolation of several polyphenols, including flavonoids, of which the most abundant were apigenin (**14**), luteolin (**15**), and luteolin-7-*O*-glucoside (**16**). Quercetin-3-rutinoside (rutin,** 2**), quercetin-3-*O*-D-galactopyranoside (hyperoside,** 3**), 3,5,7,4-tetrahydroxyflavonol (kaempferol,** 4**), 3,5,7,3,4-pentahydroxyflavone (quercetin,** 5**), and 3,5,3,4-tetrahydroxy-7-methoxyflavonol (rhamnetin,** 6**) were also present, while naringenin (**17**) was present only in trace amounts, in agreement with the data that Beara et al. (2012) reported for methanol extracts (Figure 1) [11]. Other phenolic compounds with lower molecular weights were identified as vanillic (**10**) and gallic (**1**) acids, and three derivatives of the latter: 3-*O*-galloyl-4,6-hexahydroxydiphenoyl-D-glucose (**7**), 2,3-di-*O*-galloyl-D-glucose (**8**), and 1,2,3-tri-*O*-galloyl-D-glucose (**9**), which was the most abundant [23]. Furthermore, a significant amount of cinnamic acids including cinnamic (**11**), caffeic (**12**) [11], and chlorogenic (**13**) acids was measured (Figure 2).

### 3.2. *In Vitro* Antimicrobial and Biological Assays

An infusion of dried flowers and leaves was used to assess the* in vitro* antimicrobial activity of* P. lanceolata*, expressed in terms of minimum inhibitory concentration (MIC) and minimum bactericidal concentration (MBC) (Table 3). Strains belonging to several species of* Streptococcus*, namely,* S. bovis*,* S. mitis*,* S. sobrinus*,* S. mutans*,* S. parasanguinis*, and* S. viridans* and one strain of* L. casei*, that were isolated from clinical specimens were selected for the bioassay. The infused* P. lanceolata* was assayed at concentrations ranging from 4 to 0.025 mg/mL. The susceptibility of the strains to the tea varied considerably; however, concentrations between 250 *μ*g/mL and 2.0 mg/mL showed antimicrobial activity, affecting the viability of all bacteria tested. As far as it concerns the effects on human cell lines of* P. lanceolata *infusion, we have found that the viability of both cell lines used, namely TK6 and U937 incubated in the presence of extract concentrations up to 4 mg/mL does not decrease in comparison with the respective controls.

### 3.3. *In Vivo* Assessment of Antimicrobial Activity of* P. lanceolata* Infusions

For the test group the differences in streptococci concentration (CFU/mL) between T0 and T1 and between T0 and T2 were significant (*P* < 0.001 for both); the difference between T1 and T2 was not significant. The differences in lactobacilli concentration (CFU/mL) were not significant between any two time points.

For the control group the differences in the density (CFU/mL) of streptococci and lactobacilli were not significant.

The difference in the streptococci density between test and control groups was not significant at T0 but was significant at T1 and T2, whereas the differences in the lactobacilli concentration were not significant (Table 4).

## 4. Discussion

*P. lanceolata* infusions demonstrated good* in vitro* antimicrobial activity, particularly against streptococci. The biocide potential of some species of the genus* Plantago* is well known:* P. major* extracts, for example, caused strong alterations in the cell wall structure of different gram positive bacteria [24]. Also* P. lanceolata* extracts in different solvents exhibited mild antimicrobial activity, most likely due to the presence of flavonoids and terpenes [25]. Interestingly, the water extracts of* P. lanceolata* produced the best antimicrobial effects, exhibiting weak or moderate antimicrobial activity against* S. aureus*,* S. epidermidis*,* S. marcescens*, and* Proteus vulgaris* [26]. Moreover, Bazzaz and Haririzadeh (2003) reported that the pressed juice of fresh* P. lanceolata* has a bactericidal effect [27].

The* clinical* test demonstrated that subjects who used a* P*.* lanceolata* mouth rinse presented a significant decrease in streptococci compared to subjects treated with the placebo, whereas only a slight (not significant) decrease was observed for lactobacilli counts following the treatment.

Apart from the strain-specific differences, a possible explanation for the poor reduction of lactobacilli with respect to streptococci is that the latter normally grows on exposed surfaces that are easily accessible, whereas lactobacilli and related species recover in shed retentive areas [28] that have limited contact with the mouth rinse.

Furthermore, in the present study, only the short-term (seven days) effect of* P. lanceolata* against oral streptococci and lactobacilli was assessed* in vivo*. The promising results we have got suggest that further studies need to be undertaken to establish whether the capability of reducing mutans streptococci salivary counts will be long lasting, and whether no resistance will occur. In addition, the long-term patient acceptability and compliance need to be assessed.

The phenolic content and the major phenolic compounds of the whole plant infusion, as well as that of isolated leaves, flowers, and roots of* P. lanceolata*, were determined. The results of analysis showed that all extracts have a high phenolic content. Nine flavonoids, three cinnamic acids, two compounds with a C6-C1 skeleton, and three of their glycosylated derivatives were identified. In particular, the leaf extract is the richest in polyphenols, followed by that of the flowers and, to a lesser extent, the roots. A significant difference in the flavonoid content was also detected, with the highest flavonoid content occurring in the flower extract, followed by the leaves, whereas none was detected in the roots. In accordance with previous studies on* P*.* lanceolata* phenolic constituents [11], our analyses also detected the presence of cinnamic acids in all extracts, but most prevalently in the leaves. The content of coumarins was lower than the other phenolics, however, and constant in the three types of extracts. In contrast, the lipid content was markedly low and only present in the root extract. Finally, no lignans were found in any of the extracts that were analysed. The antistreptococcal activity of* P. lanceolata* infusions could be related to the phenolic substances occurring in the infusion and is most likely the result of the synergistic activity of the many phenolics that were isolated. Chlorogenic and caffeic acids inhibit the growth of* S. mutans* [29], and the presence of significant concentrations of apigenin, an effective inhibitor of GTFs from* S. mutans* and* S. sanguinis*, is also noteworthy. The adherence of bacterial cells to the tooth surface is important for the development of carious lesions, and interference with the mechanisms of adherence can prevent dental caries. Furthermore, as suggested by Özan et al., the phenolic substances found in plant extracts most likely act on the microbial membrane or the surface of the cell wall, causing structural and functional damage [30]. However, further research is needed to identify which pure compound could be responsible for the antibacterial properties and to determine its potential for use in pharmaceutical industries.

*P*.* lanceolata* mouth rinse might offer a new option for the microbiological control of dental caries because the infusion is easily obtained and inexpensive and exhibits beneficial effects. There is an urgent need for alternative prevention and treatment options that are safe, effective, and economical, given the incidence of oral disease and the adverse effects (as tooth and tongue discoloration and taste disorder) of certain antibacterial agents that are currently used in dentistry.

Based on these considerations, a* P*.* lanceolata* infusion might be useful as an ancillary measure to control the proliferation of oral cariogenic flora. Further studies, particularly* in vivo* and* in situ* studies, are needed to establish conclusive evidence of the effectiveness of* P. lanceolata* extract against dental caries, either alone or in combination with conventional therapies, for improving oral health.

## Figures and Tables

**Table 1:** Average amount of the main classes of compounds in the infusion of the whole* P. lanceolata* plant.

Phytochemical	Average amount (*μ*g/g of DW)
Flavonoids	358 ± 20
Coumarins	9 ± 0.5
Lipids	1120
Cinnamic acid content	200
Lignans	—
Phenolic content	1368 ± 54

**Table 2:** Results of the phytochemical analysis of the infusion of flowers, leaves, and roots of *P. lanceolata*.

Phytochemical	Flowers	Leaves	Roots
Flavonoids	+++	++	—
Coumarins	+	+	+
Lipids	—	—	+
Cinnamic acid content	++	+++	+
Lignans	—	—	—
Phenolic content	++	+++	+

*Key*: +++: high concentration, ++: medium concentration, +: low concentration, —: absent.

**Table 3:** *In vitro* antimicrobial activity of *P. lanceolata*, expressed in terms of minimum inhibitory concentration (MIC) and minimum bactericidal concentration (MBC).

Species	MIC90 (mg/mL)	MBC (mg/mL)
*Streptococcus bovis *	0.50	1
*Streptococcus mitis *	0.25	0.50
*Streptococcus mutans *	0.50	1
*Streptococcus sobrinus *	0.50	1
*Streptococcus parasanguinis *	1	2
*Streptococcus viridans *	0.50	0.50
*Lactobacillus casei *	0.75	2

**Table 4:** Density of the *Streptococci mutans* CFU (> or = 10^5^ CFU/mL) and *Lactobacilli casei* CFU (> or = 10^5^ CFU/mL) at T0, T1, and T2.

	Streptococci		Lactobacilli	
	Test	Control	Test	Control
T0	85.7%	85.7%	85%	90%
T1	35.7%	78.6%	60%	80%
T2	28.6%	85.7%	65%	75%
